# What the Recommendations of the Community Health Promotion Expert Panel Mean to NCCDPHP Divisions and Programs

**Published:** 2007-06-15

**Authors:** Brick Lancaster, Lynda A Anderson

**Affiliations:** Office on Smoking and Health, National Center for Chronic Disease Prevention and Health Promotion, Centers for Disease Control and Prevention; Health Care and Aging Studies Branch, National Center for Chronic Disease Prevention and Health Promotion, Centers for Disease Control and Prevention, Atlanta, Ga

It was our pleasure to review and respond to the *Recommendations for Future Efforts in Community Health Promotion: Report of the National Expert Panel on Community Health Promotion* (1). Although we cannot speak for all the divisions and programs within the Centers for Disease Control and Prevention's (CDC's) National Center for Chronic Disease Prevention and Health Promotion (NCCDPHP), we both have had more than 10 years of experience working in the broad areas of community health promotion at various levels in NCCDPHP. We were grateful to be asked to be observers of the discussions among members of the expert panel. Reading the recommendations caused us to recall important discussions that occurred in the 1990s concerning health education and research as well as community capacity related to community health promotion.

The expert panel's recommendations provide a framework that NCCDPHP divisions and programs can use to review, plan, implement, and evaluate community health promotion programs. The cross-cutting nature of the recommendations provides an important impetus for collaboration across programmatic and research areas. To benefit from these recommendations to the greatest extent possible, we in public health cannot lose sight of issues such as program goals and priorities, how programs work together, the focus of national programs at state or community levels, existing funding streams, potential flexibility in use of funds, and the partnerships necessary for effective community health promotion. NCCDPHP is determining how best to address these issues and how it will influence the delivery of community health promotion and chronic disease prevention programs in the future, especially how these issues will affect funding and program organization at state and local levels.

A key recommendation of the expert panel deals with closing the gap between discovery and practice (i.e., the discovery of innovative model public health programs and their wide adoption in community practice) (Recommendation 4). This recommendation should resonate across programs throughout NCCDPHP. Moving research findings into community practice is an issue we in public health are all grappling with — whether in determining what constitutes the "evidence base" for a set of strategies or addressing issues related to program implementation. Meeting this challenge is particularly daunting considering the growing demands for accountability and increased calls to expand the reach of effective programs to state and national levels. Thus we focus the rest of this brief commentary on how to shorten the time between the discovery of innovations and their use in community practice.

A glance at the history of public health reminds us that there is often a long latency period between the development and the widespread adoption of strategies shown to improve health (2). Clearly, we in public health have an obligation to those we serve to ensure that our science base is credible. At the same time, we must accelerate the translation of research findings into public health practice (3,4).

To do so, however, we first must address three questions. First, do public health practitioners and their partners use the same vocabulary and definitions for "evidence-based" and "research translation"? Clearly, a common language would make it easier to discuss, describe, identify, and share effective strategies and tools for programs. The conveners of the expert panel and the NCCDPHP leadership have begun to develop consensus definitions for these key terms. An inclusive, ongoing dialogue among program leaders will not only foster stronger collaborations but can create a common language around research translation. The second question we need to address is, who will provide the leadership for the follow-up on these recommendations? The answer to this question is particularly important as programs address issues such as what constitutes "credible evidence" and what steps are involved in the research translation process, including developing and testing theories, identifying principles defining program effectiveness, replicating findings in other settings, determining technical assistance needs and requirements, finding a balance between agency and community input, and developing implementation and evaluation plans. The third question is, how will we in public health effectively engage partners, including state health departments, in this research translation process? Throughout its history, NCCDPHP has developed and nurtured partnerships with numerous public and private entities. These partnerships substantially improve and expand the scope and depth of public health activities. At the same time, these partnerships challenge us to think through how to effectively engage partners and respond to their diverse missions and perspectives so that we can help them strategically enhance their programs without damaging their core program initiatives.

The first meeting of the expert panel and the formation of an NCCDPHP ad hoc committee are a good start, but these efforts require continued engagement of partners, both internal and external, to make the panel's recommendations a reality. It will be important to create mechanisms for continued sharing of perspectives, experiences, and goals across NCCDPHP programs and with partners. We encourage the center and its constituent divisions and programs to take a proactive stance and work together to close the chasm between research and practice. We encourage NCCDPHP divisions to review the recommendations of the expert panel and continue to communicate with staff members and participate in center-wide follow-up activities related to issues raised by the panel's broader recommendations.
